# Long-Term Dietary Nitrate Supplementation Does Not Prevent Development of the Metabolic Syndrome in Mice Fed a High-Fat Diet

**DOI:** 10.1155/2018/7969750

**Published:** 2018-08-05

**Authors:** V. B. Matthews, R. Hollingshead, H. Koch, K. D. Croft, N. C. Ward

**Affiliations:** ^1^School of Biomedical Sciences, The University of Western Australia, Perth, WA, Australia; ^2^School of Biomedical Sciences & Curtin Health Innovation Research Institute, Curtin University, Perth, WA, Australia; ^3^Medical School, The University of Western Australia, Perth, WA, Australia

## Abstract

**Background:**

Nitric oxide (NO) is an important vascular signaling molecule that plays a role in vascular homeostasis. A reduction in NO bioavailability is thought to contribute to endothelial dysfunction, an early risk factor for both cardiovascular disease and type 2 diabetes. Dietary nitrate, through the nitrate-nitrite-NO pathway, may provide an alternate source of NO when the endogenous eNOS system is compromised. In addition to a role in the vascular system, NO may also play a role in the metabolic syndrome including obesity and glucose tolerance.

**Aim:**

To investigate the effect of long-term dietary nitrate supplementation on development of the metabolic syndrome in mice fed a high-fat diet.

**Methods:**

Following 1 week of acclimatisation, male (6–8 weeks) C57BL6 mice were randomly assigned to the following groups (10/group) for 12 weeks: (i) normal chow + NaCl (1 mmol/kg/day), (ii) normal chow + NaNO_3_ (1 mmol/kg/day), (iii) high-fat diet + NaCl (1 mmol/kg/day), and (iv) high-fat diet + NaNO_3_ (1 mmol/kg/day). Body weight and food consumption were monitored weekly. A subset of mice (5/group) underwent running wheel assessment. At the end of the treatment period, all mice underwent fasting glucose tolerance testing. Caecum contents, serum, and tissues (liver, skeletal muscle, white and brown adipose, and kidney) were collected, frozen, and stored at −80°C until analysis.

**Results:**

Consumption of the high-fat diet resulted in significantly greater weight gain that was not affected by dietary nitrate. Mice on the high-fat diet also had impaired glucose tolerance that was not affected by dietary nitrate. There was no difference in adipose tissue expression of thermogenic proteins or energy expenditure as assessed by the running wheel activity. Mice on the high-fat diet and those receiving dietary nitrate had reduced caecum concentrations of both butyrate and propionate.

**Conclusions:**

Dietary nitrate does not prevent development of the metabolic syndrome in mice fed a high-fat diet. This may be due, in part due, to reductions in the concentration of important short-chain fatty acids.

## 1. Introduction

Nitric oxide (NO) is an important vascular signaling molecule that plays a major role in the control of vascular function and tone [1]. The majority of the body's NO is synthesised endogenously through conversion of L-arginine to citrulline by endothelial cell-derived nitric oxide synthase (eNOS). Once released, NO diffuses to the underlying smooth muscle cells and stimulates the production of cyclic guanosine monophosphate (cGMP) and subsequent relaxation of the vessel wall. A reduction in the bioactivity and/or bioavailability of endogenously-derived NO is thought to be the main cause of endothelial dysfunction, an early risk factor for cardiovascular disease (CVD) and type 2 diabetes (T2DM) [2].

There is substantial epidemiological evidence to suggest that diets rich in fruits and vegetables have beneficial effects on CVD and its risk factors [3]. Dietary nitrate (NO_3_^−^), found predominantly in green leafy vegetables, may be one of the beneficial components of such a diet as it represents an alternative source of NO. Dietary nitrate is well absorbed, and approximately 25% of ingested nitrate is secreted into the saliva and 20% of this (~5% of ingested nitrate) is converted to nitrite (NO_2_^−^) by bacteria in the mouth. The nitrite is then swallowed and absorbed where it can be stored to act as a pool of NO or have direct vasodilatory effects [4]. This nitrate/nitrite-derived NO pool represents a NOS-independent pathway that can be used to supplement endogenous NO supplies or replace them when they are compromised.

The metabolic syndrome is classified as a cluster of risk factors that include hypertension, obesity, impaired glucose/insulin tolerance, and dyslipidaemia, and is a significant contributor to the development of both CVD and type 2 diabetes [5]. Endothelial dysfunction is often present in the metabolic syndrome, and studies have shown that reduced NO production and/or bioavailability has a role in the pathogenesis of many risk factors associated with the condition [6]. We have previously shown that low and moderate dose dietary nitrate can prevent endothelial dysfunction and improve plaque composition and stability in the ApoE^−/−^ mouse [7]. Dietary nitrate has also been shown to reduce triglycerides and improve intravenous glucose tolerance in eNOS-deficient mice [8]. Additionally, nitrate has been shown to induce antiobesity effects, including weight loss and reductions in body fat, via increases in the expression of thermogenic genes in brown adipose tissue [9]. Despite this, there is little evidence examining the effect of long-term dietary nitrate supplementation on the development of features of the metabolic syndrome. Therefore, the aim of the present study was to investigate the effect of long-term dietary nitrate supplementation on weight gain, glucose and insulin tolerance, and indices of thermogenesis in C57BL6 mice fed a high-fat diet.

## 2. Methods

### 2.1. Animal Study

Male C57BL6 mice (6–8) weeks were purchased from the Animal Resource Centre (Perth, Australia) and maintained at 23 ± 2°C under a 12-hour light–dark cycle. Following a week of acclimatisation, the mice were randomly divided into one of four groups (*n* = 10, 5 mice/cage): (i) normal chow + NaCl-supplemented water (1 mmol/kg/day), (ii) normal chow + NaNO_3_-supplemented water, (iii) high-fat diet + NaCl-supplemented water (1 mmol/kg/day), and (iv) high-fat diet + NaNO_3_-supplemented water (1 mmol/kg/day). The normal chow diet was commercial rodent chow consisting of 4.8% wt/wt fat, while the high-fat diet (HFD) contained 23.5% wt/wt fat (clarified butter). The nitrate dose is both physiologically relevant and comparable to the dose previously used where we observed beneficial effects on both vascular function and atherosclerotic plaque composition [7]. Mice were allowed ad libitum access to water and food, with all diets prepared by Specialty Feeds (Glenn Forrest, Australia). The level of nitrate and nitrite in the food pellet was 9.9 *μ*g/g and 0.8 *μ*g/g, respectively [7]. The mice were maintained on their respective diets for 12 weeks. Body weight and food intake were measured weekly. The study was approved by the Royal Perth Hospital Animal Ethics Committee (R534/17-18) with reciprocal approval from Curtin University Animal Ethics Committee. All animal experiments were compliant with the National Health and Medical Research Council (NHMRC) guidelines for the Care and Use of Laboratory Animals in Australia.

### 2.2. Glucose Tolerance Tests

Intraperitoneal glucose tolerance tests (IPGTT) were performed on fasting (5 hr) mice at week 11. To measure blood glucose levels, blood samples were taken from the tail of fasting (5 hr) mice before (*t* = 0 min) and at subsequent time intervals of *t* = 15, 30, 45, 60, 90, and 120 min following intraperitoneal administration of 1 g glucose/kg. Blood glucose levels were measured using Accu-Chek Performa Strips and Glucometer (Roche Diagnostics, Australia). The area under the curves (AUCs) was calculated using the trapezoidal method.

### 2.3. Tissue Histology and Pathology

Fasted (5 hr) mice were anaesthetised with methoxyflurane at week 12, and liver and white adipose tissues were collected and fixed in 4% formaldehyde overnight before being incubated in 50% ethanol and embedded in paraffin. The tissues were then cut into 4 *μ*m sections and stained with hematoxylin and eosin (CellCentral, UWA) before being visualized and photographed using a Nikon Eclipse TS100 microscope. Additional liver, white and brown adipose tissues, gastrointestinal tract, kidney, and skeletal muscle were collected and snap frozen in liquid nitrogen and stored at −80°C until analysis. White and brown adipose tissue protein expression of AMPK (Cell Signaling, USA), phosphorylated AMPK (Cell Signaling, USA), ACC (Cell Signaling, USA), phosphorylated ACC (Cell Signaling, USA), and UCP-1 (Cell Signaling, USA) were determined using western blot and normalised to actin (Sigma, USA), as previously described [7]. In a subset of mice (5/group), kidneys were also collected and stained with antisodium glucose cotransporter 2 (SGLT2, Santa Cruz Biotechnology) as previously described [10].

### 2.4. Energy Expenditure

Subsets of mice (5/treatment group) were housed individually with wireless running wheels during week 10 of the treatment period. Each mouse was allowed free access to their running wheel, which collected data on behaviour and activity, assessed as wheel revolutions and distance covered by the mouse using the Running Wheel Manager Acquisition Software (Med Associates Inc.). Individual mice were housed with their wheel for a period of 6 days. The first 3 days were an acclimatisation period with all measurements discarded from final analysis. The final 3 days of activity were collected and averaged using the Wheel Analysis Software (Med Associates Inc.) to provide information on daily activity level and mean overall activity level.

### 2.5. Caecum Short-Chain Fatty Acid (SCFA) Analysis

Caecum samples isolated from the gastrointestinal tract were collected and snap frozen in liquid nitrogen and stored at −80°C until analysis. SCFA concentrations of acetate, butyrate, and propionate were analysed using gas chromatography–mass spectrometry as previously described [11].

### 2.6. Serum Nitrate/Nitrite Concentrations

Fasted (5 hr) mice were anaesthetised with methoxyflurane at week 12, and blood samples were obtained via cardiac puncture. Serum was separated via centrifugation (3000 rpm, 4°C, 10 mins) and stored at −80°C until analysis. Nitrate and nitrite concentrations were determined using gas chromatography–mass spectrometry (GC–MS) as previously described [12].

### 2.7. Statistical Analysis

Statistical analysis was performed using SPSS (version 21.0). All values are expressed as mean ± standard error (SEM). Data was analysed using repeated measures analysis of variance (ANOVA) and one-way ANOVA with Tukey post hoc analysis to determine differences between groups. A *p* < 0.05 was determined to be of statistical significance. The data used to support the findings of this study are available from the corresponding author upon request.

## 3. Results

### 3.1. Nitrate Diet and the Effect of Dietary Nitrate on Obesity, Hepatic Fat, and Food Intake

**Figure 1:**
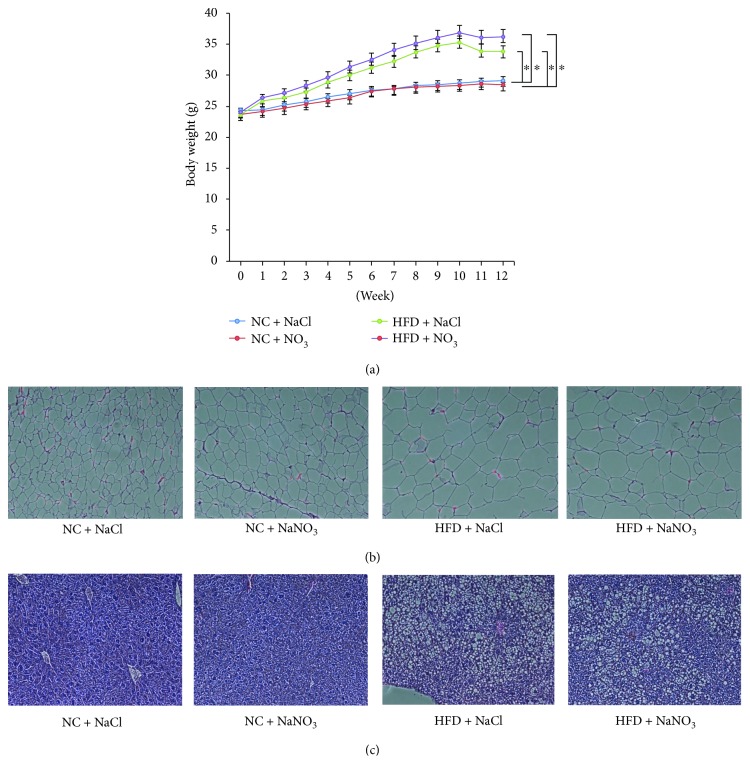
(a) Body weight gain, (b) adipocyte hypertrophy, and (c) hepatic lipid droplet accumulation in C57BL6 mice fed a normal chow or HFD with or without dietary nitrate for 12 weeks. *n* = 10/group, ANOVA (^∗^*p* < 0.05).

Previous studies suggest mice consume approximately 5 g of chow and 6 mL of water per day [13]. Our previous nitrate study demonstrated that water supplemented with 1 mmol of nitrate contains 130 *μ*g/mL of nitrate and 3 *μ*g/mL of nitrite [7]. In the present study, mice consuming the HFD gained significantly more weight over 12 weeks compared to mice consuming the normal chow diet. The addition of nitrate had no effect on weight gain when added to either the normal chow or HFD (Figure 1(a)). Average food consumption (g/mouse/day) was lower in mice consuming the HFD compared to normal chow. When measured as calories/mouse/day, mice fed the HFD consumed more than those fed the normal chow diet. Neither of these reached statistical significance, and dietary nitrate supplementation had no effect on food consumption with either diet (data not shown).

There was noticeable adipocyte hypertrophy in mice consuming the HFD compared to normal chow; however, addition of dietary nitrate had no effect on this (Figure 1(b)). Hepatic fat accumulation as evidenced by lipid droplets was also higher in mice consuming the HFD compared to normal chow, and once again, this was not affected by dietary nitrate (Figure 1(c)).

### 3.2. The Effect of Dietary Nitrate on the Development of Diet-Induced Glucose Intolerance

**Figure 2:**
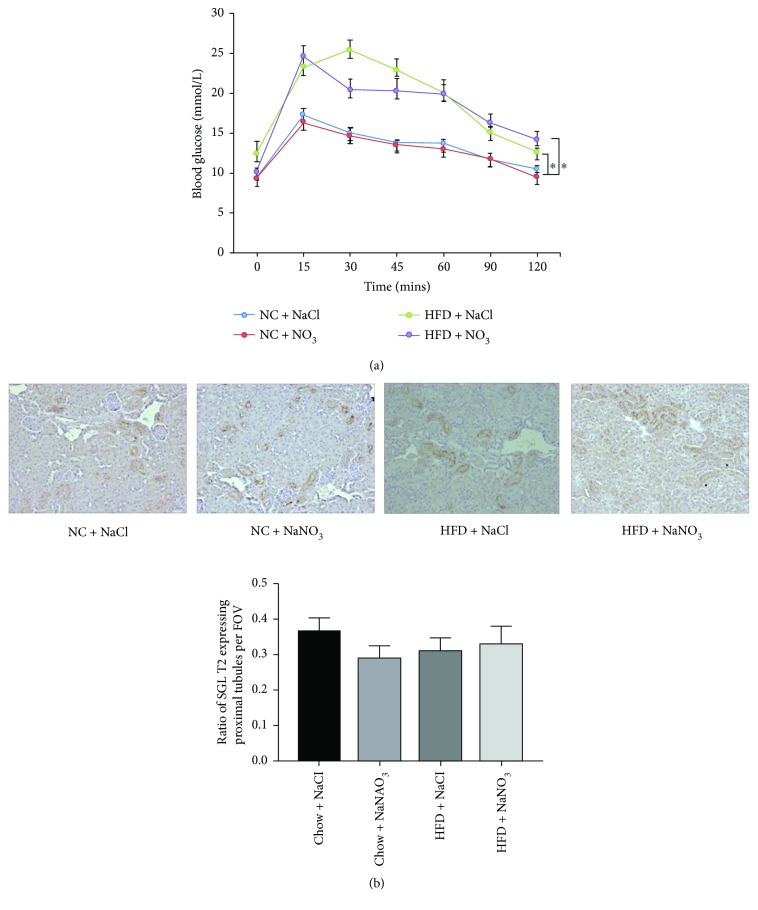
(a) Fasting glucose tolerance testing in C57BL6 mice fed a normal chow or HFD with or without dietary nitrate for 12 weeks. *n* = 10/group, ANOVA (^∗^*p* < 0.05). (b) Representative immunohistochemistry images (depicted by brown staining, magnification at 200×) and the ratio of renal proximal tubule expression of SGLT2 in C57BL6 mice fed a normal chow or HFD with or without dietary nitrate for 12 weeks (*n* = 5/group; FOV: field of view).

Mice fed the HFD for 12 weeks had significantly impaired glucose tolerance compared to mice receiving the normal chow diet. Addition of dietary nitrate had no significant effect on glucose tolerance with either diet (Figure 2(a)). As SGLT2 is responsible for glucose reabsorption and may influence blood glucose levels, we assessed SGLT2 expression in the renal proximal tubules in a subset of mice (5/group). We observed no difference in renal proximal tubule SGLT2 expression, regardless of the diet they were receiving (Figure 2(b)).

### 3.3. The Effect of Dietary Nitrate on Markers of Metabolic Activity and Energy Expenditure

There was no significant difference in white or brown adipose tissue expression of AMPK, p-AMPK, ACC, or p-ACC in mice fed the normal chow or HFD, with or without the addition of nitrate (data not shown). UCP1 expression was not detectable in white adipose tissue in any of the dietary treatment groups (data not shown) and was not significantly different in brown adipose tissue between the normal chow and HFD, with or without nitrate (data not shown).

Energy expenditure as assessed via running wheel use revealed no significant difference between any of the dietary treatment groups in the amount of exercise as assessed via the total distance or the total revolutions over a 3-day period (Table 1).

### 3.4. The Effect of Dietary Nitrate Supplementation on Circulating Nitrate and Nitrite

**Figure 3:**
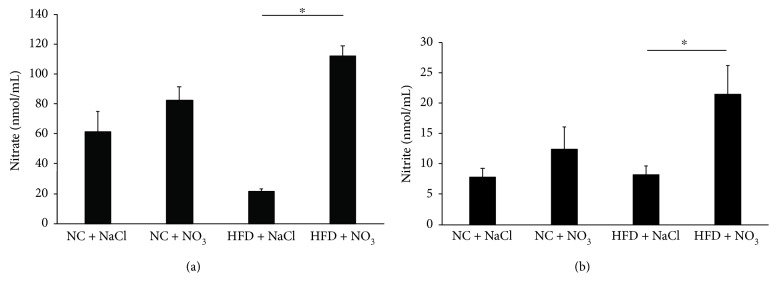
Serum (a) nitrate and (b) nitrite concentrations in C57BL6 mice fed a normal chow or HFD with or without dietary nitrate for 12 weeks. *n* = 10/group, ANOVA (^∗^*p* < 0.05).

Circulating serum nitrate and nitrite levels were increased in mice receiving the nitrate supplemented diet, and this appeared most pronounced and significant in the HFD group (Figures 3(a) and 3(b)).

### 3.5. The Effect of Dietary Nitrate on Production of Short-Chain Fatty Acids

**Figure 4:**
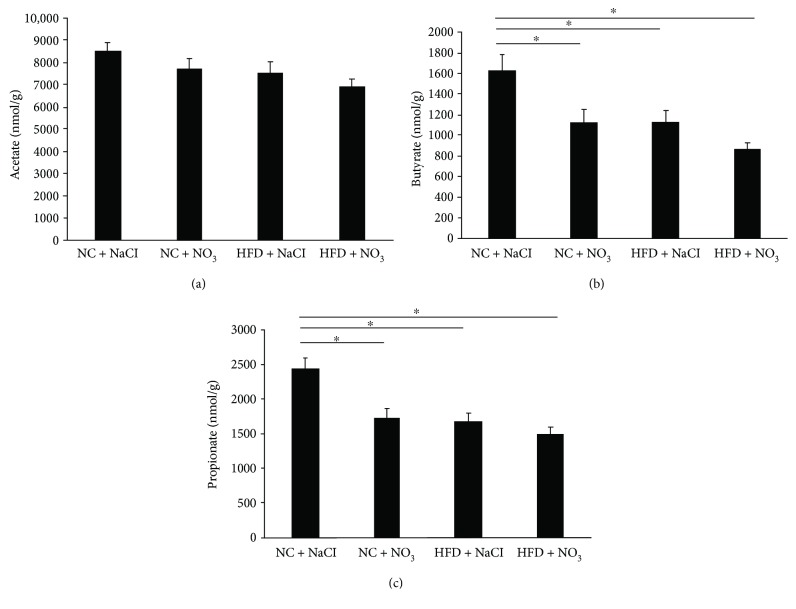
Caecum (a) acetate, (b) butyrate, and (c) propionate concentrations in C57BL6 mice fed a normal chow or HFD with or without dietary nitrate for 12 weeks. *n* = 10/group, ANOVA (^∗^*p* < 0.05).

There was no effect of any dietary treatment on caecum levels of acetate (Figure 4(a)). Caecum concentrations of butyrate and propionate were significantly reduced in the nitrate-supplemented normal chow and both the HFD and nitrate-supplemented HFD compared to normal chow diet alone (Figures 4(b) and 4(c)).

## 4. Discussion

Inorganic nitrate was originally believed to be an inert by-product of NO metabolism that was readily excreted by the body but could be potentially toxic and carcinogenic when given in supraphysiological doses [14]. However, nitrate is present in the diet, particularly green leafy vegetables [15], and discovery of the nitrate-nitrite-NO conversion pathway in mammals [16] has highlighted the possibility of using this system to enhance or supplement the body's NO supplies. This may be particularly important in disease states where NO levels are compromised, such as CVD and T2DM. Inorganic nitrate has previously been shown to improve risk factors for CVD such as endothelial dysfunction and blood pressure in humans [17], protect against myocardial ischaemia-reperfusion injury in mice [18], reverse features of the metabolic syndrome in the eNOS knockout mouse [8] and mice having undergone ovariectomy [19], prevent endothelial dysfunction, and improve plaque composition and stability in the ApoE^−/−^ mouse [7].

In the present study, we examined the potential for long-term dietary nitrate supplementation to prevent development of the metabolic syndrome in the C57BL6 mouse fed a high-fat diet for 12 weeks. Despite seeing significant increases in circulating nitrate and nitrite in mice receiving the nitrate-supplemented diets, this did not result in improvements in any features of the metabolic syndrome. Mice on the HFD gained more weight and had significant glucose intolerance compared to their normal chow-fed counterparts, and addition of nitrate to their diet did not ameliorate this. Furthermore, there was no effect of dietary nitrate on white or brown adipose tissue expression of proteins involved in thermogenesis or fatty acid oxidation. Moreover, dietary nitrate significantly reduced caecum concentrations of the short-chain fatty acids, butyrate, and propionate, regardless of whether the mice were consuming a normal chow or high-fat diet.

Obesity, caused by excessive accumulation of white adipose tissue, is a major component of the metabolic syndrome. White adipose tissue is the primary site of energy storage and hormone and cytokine release. In contrast, brown adipose tissue is important for both basal and thermal energy expenditure [20]. A previous *in vitro* study has shown that cGMP promotes a healthy expansion and browning of white adipose tissue to so-called “brown-like” or “brite” cells that have been suggested to have both antiobesity and insulin-sensitising effects [21]. As cGMP is a target of NO, it is possible that dietary nitrate treatment, through conversion via the nitrate-nitrite-NO pathway, could target cGMP in adipose tissue and enhance this browning phenomenon. Through a series of *in vitro* and *in vivo* studies, Roberts et al. found out that this browning effect of nitrate was mediated through both a cGMP and protein kinase-G mediated mechanism that was further enhanced in hypoxia [9, 22]. Despite this, in the present study, we saw no beneficial effects of dietary nitrate on weight gain, adipocyte hypertrophy, or hepatic lipid accumulation, as well as no difference in markers of thermogenesis or fatty acid oxidation in the white or brown adipose tissue of these mice. While the reason for the lack of beneficial effect in our study is unknown, it is possible that differences in the dose of nitrate, mouse model, and severity of disease (vascular versus metabolic endpoints) or study duration may have all contributed. This is supported by our previous study where we saw dose-dependent effects of dietary nitrate on endothelial function and atherosclerotic plaque size and composition [7] and the known negative cross talk that exists between the nitrate-nitrite-NO and eNOS pathways [23]. It is also possible that the benefits of dietary nitrate are more apparent in a disease model that specifically targets the endogenous eNOS pathway, resulting in detrimental effects on the vasculature, as seen in the eNOS^−/−^ and ApoE^−/−^ mouse models [7, 8]. In addition, despite seeing significant increases in circulating nitrate and nitrite suggesting conversion of the dietary nitrate to nitrite by the mouse oral bacteria, subsequent tissue uptake of nitrate/nitrite may have been impaired or reduced, limiting any potential beneficial effects. While future studies with higher doses or supplementation with nitrite are warranted, in the present study, we chose to use a moderate dose of dietary nitrate, which is both physiologically relevant and represents what could be achieved through a diet rich in fruits and vegetables, in contrast to nitrite, which is predominately found in processed meats [15].

An alternate possibility for the lack of beneficial effect may be related to the reduction we observed in the caecum concentrations of the SCFAs butyrate and propionate. SCFAs are produced by the gut microbiome and have been associated with several key metabolic processes, including adiposity, food intake, lipid metabolism, glucose homeostasis, and insulin resistance [24, 25]. Gut-derived propionate is used in the hepatic synthesis of odd-chain fatty acids, which are associated with a reduced risk of T2DM [26], and butyrate supplementation has been shown to improve insulin sensitivity and increase energy expenditure in mice fed a HFD [27]. In the present study, we saw significant reductions in both propionate and butyrate in the HFD-fed mice compared to normal chow-fed mice, which support these HFD-fed mice developing features of the metabolic syndrome. Unexpectedly, however, dietary nitrate supplementation was unable to prevent this reduction in the HFD-fed mice and, in fact, significantly reduced the levels of these two important SCFA in mice fed the normal chow diet. This may partly explain the lack of beneficial effect we observed on development of the metabolic syndrome, although we acknowledge that 16S RNA sequencing and analysis to confirm gut remodeling would further support this.

In conclusion, despite previous studies suggesting that dietary nitrate supplementation has beneficial effects on risk factors associated with CVD and T2DM, we observed no beneficial effect on the development of the metabolic syndrome in mice fed a HFD for 12 weeks. Although the mechanisms for this are unknown, it may be partly attributable to reductions in the concentration of the important SCFAs butyrate and propionate. Future studies investigating different doses and duration as well as intervention after disease has been established are warranted.

## Figures and Tables

**Table 1:** Energy expenditure as assessed by running wheel distance and revolutions over a 3-day period.

	NC + NaCl	NC + NaNO_3_	HFD + NaCl	HFD + NaNO_3_
Distance (meters)				
Day 1	185 ± 44	156 ± 24	104 ± 12	91 ± 39
Day 2	198 ± 51	206 ± 41	157 ± 22	111 ± 50
Day 3	221 ± 32	267 ± 65	238 ± 26	139 ± 60
Average	206 ± 38	213 ± 48	166 ± 20	114 ± 50
Revolutions				
Day 1	502 ± 126	412 ± 63	417 ± 59	296 ± 133
Day 2	528 ± 136	547 ± 108	417 ± 59	296 ± 133
Day 3	591 ± 87	684 ± 135	633 ± 69	369 ± 159
Average	541 ± 106	548 ± 99	442 ± 52	302 ± 131

Mean ± SEM (*n* = 5/group).

## Data Availability

The data used to support the findings of this study are available from the corresponding author upon request.
